# Case Report: Upper extremity fibro-adipose vascular anomaly

**DOI:** 10.3389/fsurg.2025.1613279

**Published:** 2025-09-08

**Authors:** Yutao Wang, Xue Pang, Sihai Yu, Qingyong Guo, Qichen Fan, Kuiquan Song, Yan Sun

**Affiliations:** ^1^ Department of Peripheral Vascular Surgery, Guang'anmen Hospital Jinan Hospital, China Academy of Chinese Medical Sciences (Jinan Municipal Hospital of Traditional Chinese Medicine), Jinan, China; ^2^ Department of Colorectal Surgery, The First Affiliated Hospital of Shandong First Medical University, Jinan, China; ^3^ Department of Vascular Surgery, Shandong Provincial Hospital Affiliated to Shandong First Medical University, Jinan, China

**Keywords:** fibro-adipose vascular anomaly, vascular malformation, upper limb, case report, surgical management

## Abstract

**Background:**

Fibro-Adipose Vascular Anomaly (FAVA) is a complex vascular malformation, usually presents as painful and slow-growing masses, and occurs commonly in the lower limb.

**Methods:**

This paper describes a rare case of FAVA in the upper limb, which was successfully treated with surgery. A 26-year-old female was admitted with a painful mass in the left forearm. The MRI scan and Color Doppler ultrasound showed the mass located in the flexor carpi radialis of the left forearm.

**Results:**

The patient underwent resection of the involved muscle, and the histopathological examination confirmed the diagnosis of FAVA. She was followed up for 6 months after surgery without any relevant clinical event.

**Conclusions:**

Surgical management is an effective option for FAVA.

## Introduction

Fibro-adipose vascular anomaly (FAVA) was first proposed by Alomari et al. from the Boston Children's Hospital in 2014 (1). Its primary clinical manifestations are swelling in the affected region and pain. Some patients may present with a limb contracture. FAVA is more common among young women with the calf being the most commonly involved site, and occurrence in the upper limb is rare. Here we report a female FAVA patient with lesions in the left forearm to provide a reference for the treatment of this disease. This study protocol was approved by the Ethics Committee of Jinan Municipal Hospital of Traditional Chinese Medicine. The patient has signed informed consent forms and consented to the publication of this case report.

## Case

A 26-year-old female was admitted to the hospital with a mass on the medial aspect of her left forearm in 2018. Before that, she had a firm lump on the medial aspect of her left forearm following trauma. She experienced pain on palpation, and the pain was worsened by activity and relieved by rest. She visited a local hospital and was diagnosed with “hemangioma”. She was given injections of sclerosing agents three times with a poor outcome. Therefore, she visited our hospital for further diagnosis and treatment. Physical examination: both upper limbs had equal length and size with no significant abnormality in skin color and temperature; a firm, ill-defined, poorly mobile mass measuring 11 cm × 5 cm × 4 cm was palpable 5 cm distal to the left transverse cubital crease on the medial forearm, exhibiting marked tenderness on palpation. No murmur was heard on auscultation, and there was no thrill on palpation. Passive and active range of motion of the elbow and wrist were within normal limits, and no deformity or instability was noted. The brachial artery, radial artery, and ulnar artery were palpable. No marked abnormality was observed in muscle strength and tension of both upper limbs. The patient had previously undergone sclerotherapy at an outside hospital, which was reported to have been ineffective. However, we were unable to obtain the initial radiological images or detailed treatment records from that institution. This limitation precludes a direct comparison of the lesion's appearance before and after sclerotherapy.

The hematological test results revealed the following: the white blood cell (WBC) count was 6.32 × 10⁹/L (normal range: 4.0–10.0 × 10⁹/L), the lymphocyte percentage was 52.5% (normal range: 20%–40%), the neutrophil percentage was 37.10% (normal range: 50%–70%), the hematocrit value (HCT) was 39.2 (normal range: 38%–47%), the platelet (PLT) count was 442 × 10⁹/L (normal range: 100–400 × 10⁹/L), the prothrombin time (PT) was 10.90 s (normal range: 10–14 s), the activated partial thromboplastin time (APTT) was 29.00 s (normal range: 22–35 s), and the D-dimer level was 0.15 mg/ml (normal range: <0.55 mg/ml).

**Figure 1:**
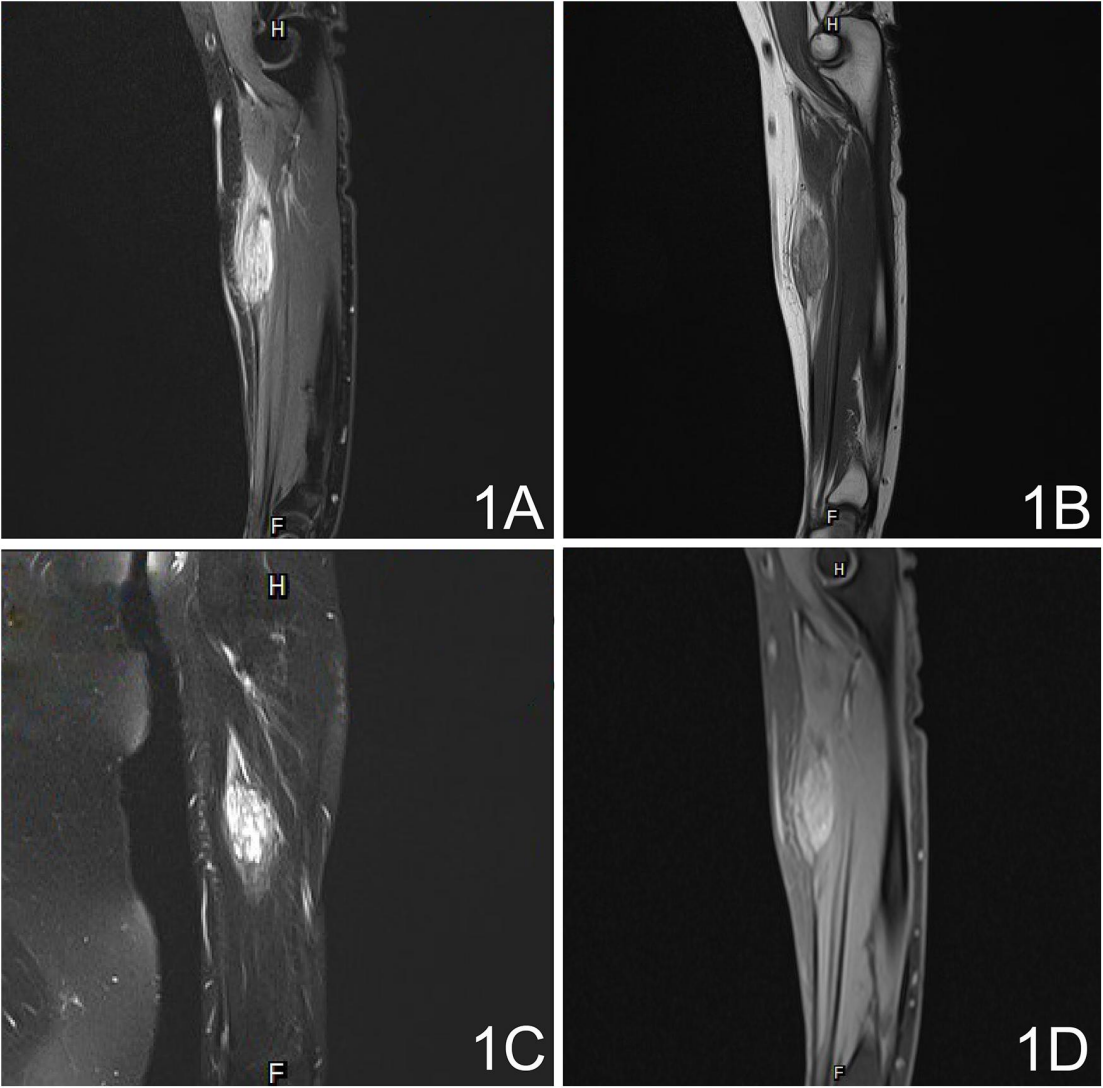
Pre-operative MRI. **(A)** Fat-suppressed T2-weighted image (FS-T2WI) shows multiple stripe-like hyperintense lesions with inhomogeneous signal. **(B)** T1-weighted image (T1WI) reveals corresponding hypointense foci. **(C)** T2-weighted image (T2WI) displays scattered small vessel cross-sections within the lesion. **(D)** Contrast-enhanced fat-suppressed T1-weighted image (CE-T1WI) demonstrates marked, heterogeneous enhancement after gadolinium injection.

**Figure 2:**
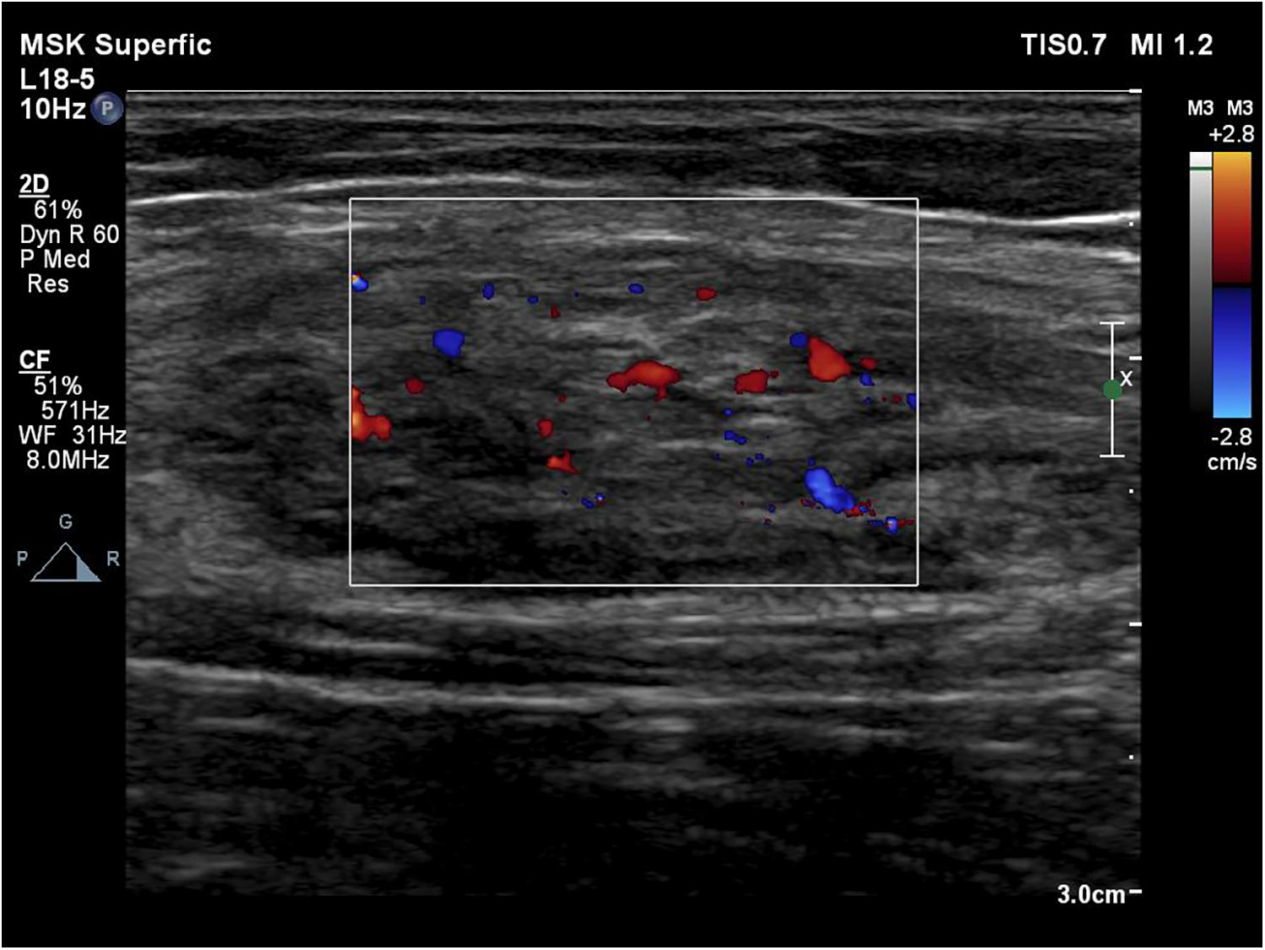
Color Doppler indicates a subcutaneous mass with inhomogeneous echogenicity with dot-like flow signals detected inside.

MRI revealed an 11 cm (length) × 5 cm (width) × 4 cm (depth) mass on the medial aspect of the left forearm. T1-weighted images (T1WI) demonstrated involvement in the flexor carpi radialis, while T2-weighted images (T2WI) showed inhomogeneous enhancement of the mass (Figure 1). Color Doppler ultrasound indicated a subcutaneous mass with inhomogeneous echogenicity, in which dot-like flow signals were detected (Figure 2).

After comprehensive pre-operative evaluation—including review of her history, clinical findings, imaging, and the poor response to sclerotherapy—no contraindications to surgery were identified. The mass was therefore excised under brachial plexus block anesthesia. During the surgery, a mass was seen located in the flexor carpi radialis of the left forearm, showing no clear demarcation with the muscle and adhesion to peripheral tissue. The flexor carpi radialis was isolated, and the muscles proximal and distal to the mass were disconnected. The mass was completely removed while carefully protecting the radial artery and veins and the superficial radial nerve branch.

**Figure 3:**
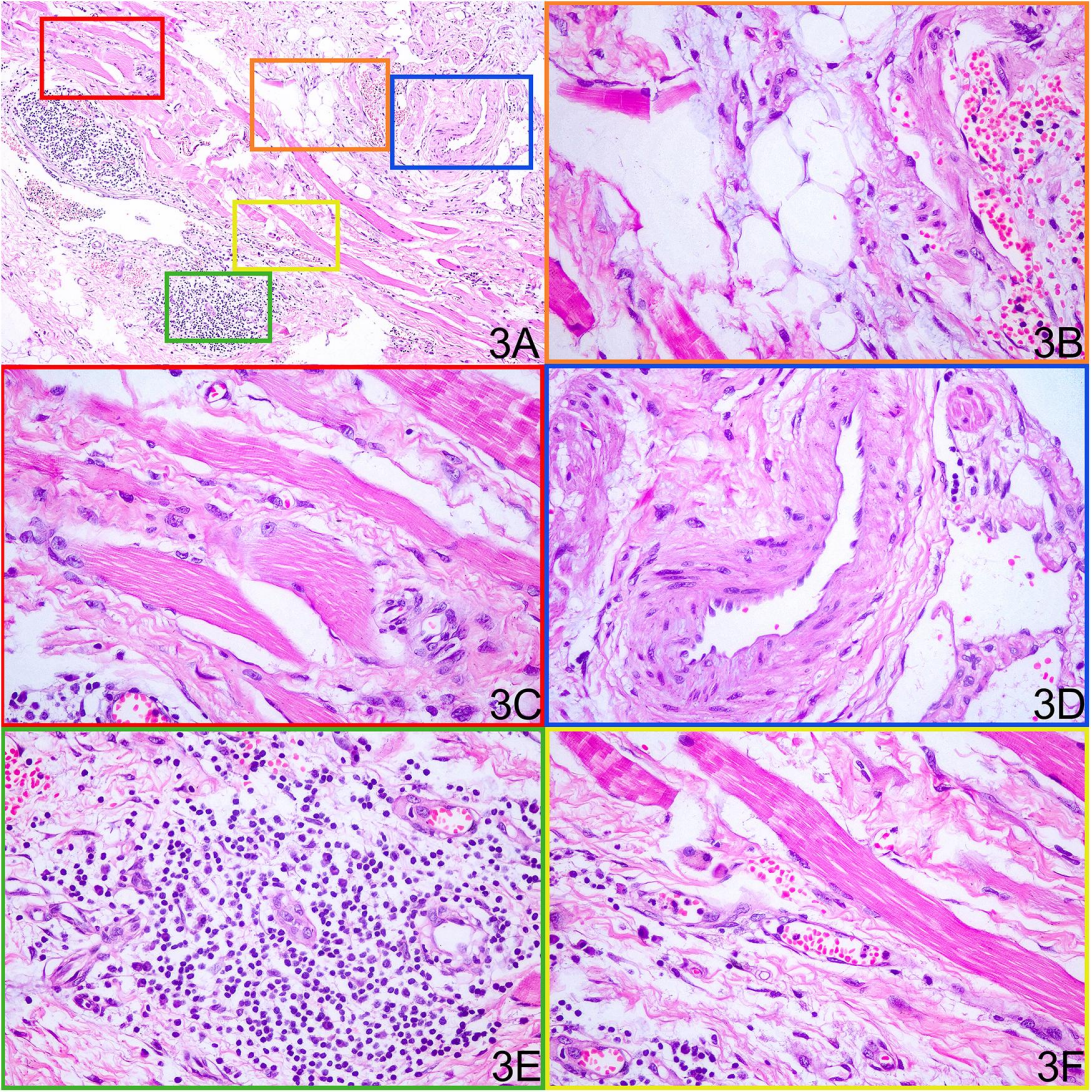
Histopathological examination of the resected tissue shows various components within the mass. **(A)** Low-power H&E overview of the resected lesion. **(B)** Mature adipocyte aggregates. **(C)** Interspersed skeletal muscle fibers. **(D)** Irregularly dilated dysplastic vessels. **(E)** Lymphocytic infiltrates. **(F)** Dense fibrous connective tissue.

**Figure 4:**
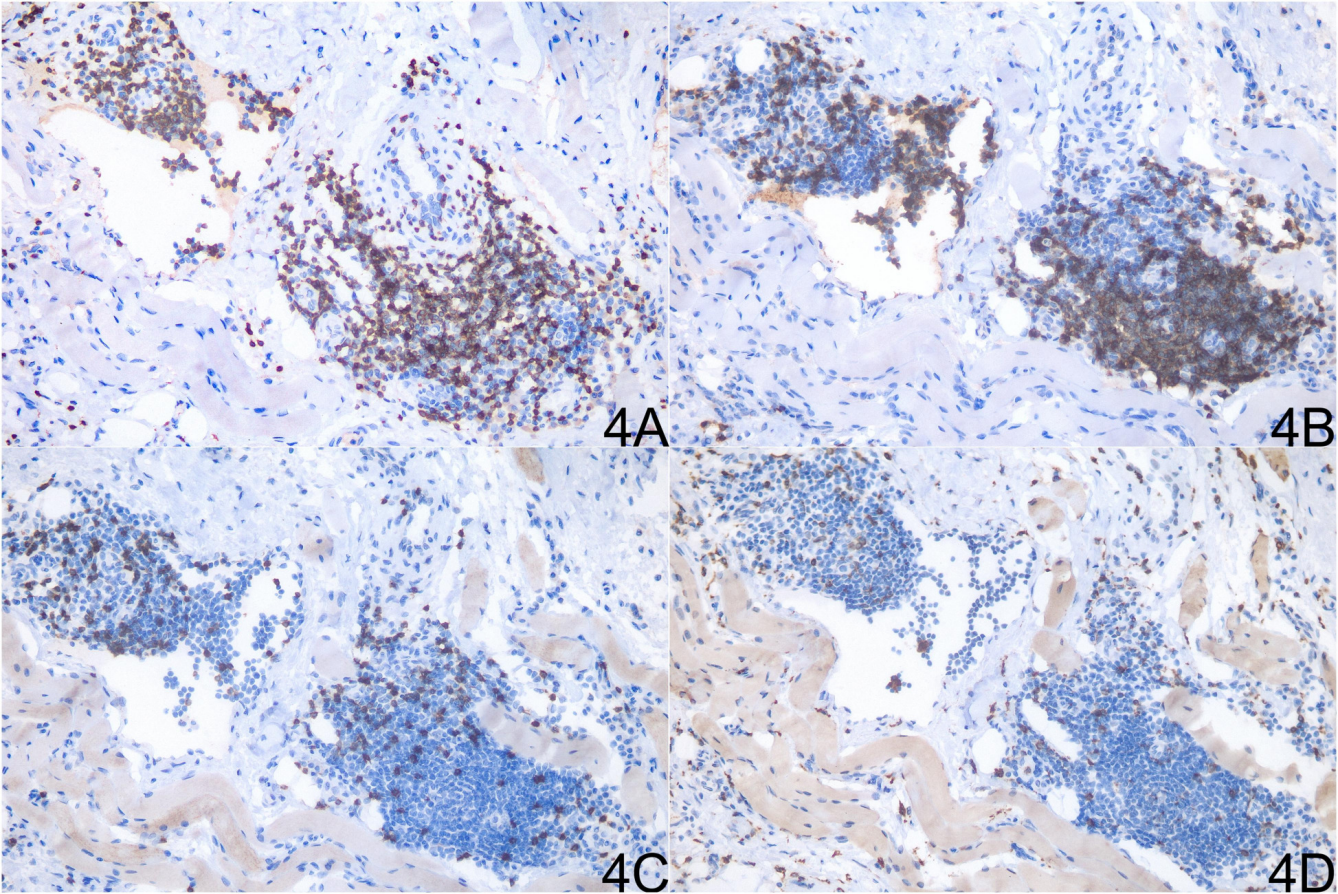
Immunohistochemical analysis of the resected tissue showing distinct lymphocyte populations. **(A)** CD3 staining. **(B)** CD20 staining. **(C)** CD8 staining. **(D)** CD68 staining.

Histopathological examination of the resected tissue revealed massive dilated vessels in the skeletal muscle, along with aggregation of adipocytes, fibrous tissue cells, and plasma cells (Figure 3). Immunohistochemical (IHC) staining of the lesion revealed the following lymphocyte populations: CD3 and CD20 staining were prominently positive, indicating a significant infiltration of T lymphocytes and B lymphocytes within the lesion. In contrast, CD8 and CD68 staining showed minimal positivity, suggesting a relatively sparse infiltration of cytotoxic T cells and histiocytes (Figure 4).

**Figure 5:**
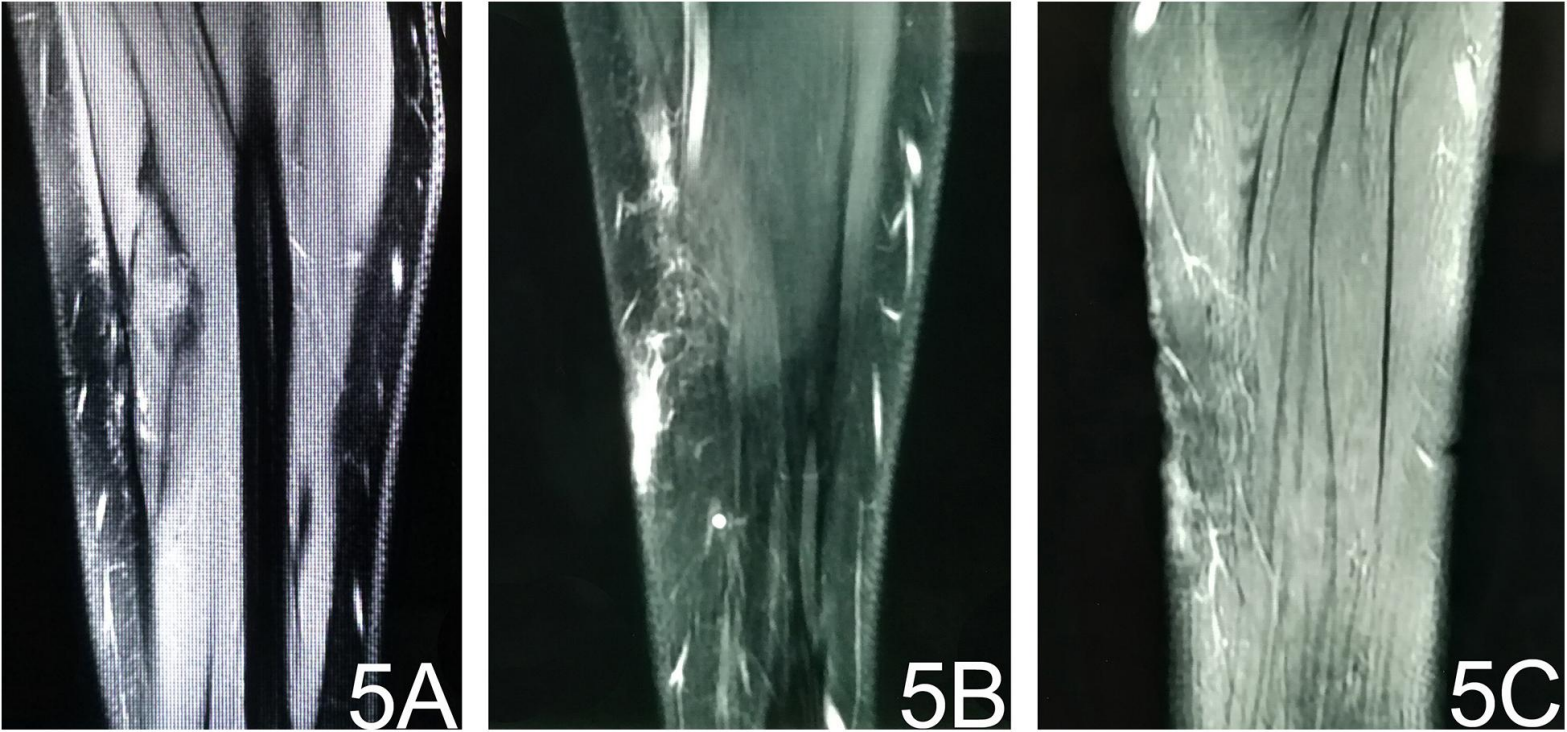
Post-operative MRI. **(A)** T1-weighted image reveals subcutaneous stripe- and patch-like hypointense areas at the operative site. **(B)** Fat-suppressed T2-weighted image shows corresponding hyperintense signal, indicating post-surgical oedema. **(C)** Contrast-enhanced fat-suppressed T1-weighted image demonstrates thin, patchy enhancement consistent with post-operative granulation tissue.

The patient was discharged on postoperative day 5 and followed up for 4 months. Repeat MRI of the left forearm showed patchy and linear long-T1/long-T2 signal areas with corresponding high signal on fat-suppressed sequences and thin, linear enhancement (Figure 5). These findings are consistent with post-operative granulation tissue and scarring; no evidence of residual or recurrent tumor was identified (Figure 5). No relevant clinical events occurred.

## Discussion

Alomari et al. (1) from the Boston Children's Hospital delved into a unique intramuscular lesion via a retrospective study in 2014. Combining clinical manifestations, radiographic images, and surgical and histopathological data, a novel subtype of vascular malformation, FAVA, was proposed. At the 22nd international conference of the International Society for the Study of Vascular Anomalies (ISSVA), FAVA was selected as the name of the newly modified category of hemangioma and vascular malformation. However, FAVA is still categorized as a tentatively unclassified disease because of inadequate understanding (2).

Alomari et al. (1) and Shaikh (3) et al. reported that FAVA is more common among female patients. There are different sites of FAVA onset, mostly in the calf, occurring most frequently in the gastrocnemius and the soleus muscles. As a feature of FAVA, pain in FAVA is different from the occasional pain caused by the venous anomaly. FAVA patients usually have persistent and acute pain after activity. In a previous study, the causes of pain in 176 patients with intramuscular venous malformations were analyzed (4), and there were 4 primary reasons for limb pain induced by intramuscular venous malformation: 1. involvement of the tendinous origin and insertion; 2. involvement of local nerves, leading to nerve thickening and increased membrane tension; 3. involvement of periosteum and cortical bone; 4. phlebolith formation. Another study reported that (5) intramuscular venous malformations lead to stronger pain intensity than subcutaneous and arthropathic lesions. This suggests that fat infiltration within the muscle fibers may lead to significant pain in FAVA, intramuscular fat infiltration disrupts muscle contraction, and muscle pain during exercise restricts voluntary limb movements. This leads to reduced movements and worsened muscle contracture, resulting in a vicious cycle. Besides muscular factors, subcutaneous focal fibrosis, local vascular lesions, and nerve involvement are non-negligible causes of pain in FAVA as well.

Beyond these macroscopic features, recent molecular studies have illuminated the role of PIK3CA mutations in FAVA pathogenesis. A significant aspect of FAVA is its association with somatic mutations in the PIK3CA gene, which encodes for a catalytic subunit of phosphatidylinositol-3-kinase (PI3K). These mutations are believed to activate the mammalian target of rapamycin (mTOR) signaling pathway, leading to excessive angiogenesis and lymphangiogenesis (6, 7). Studies have reported that approximately 62.5% of FAVA cases exhibit PIK3CA mutations, suggesting a potential role in the pathogenesis of this anomaly (8). However, not all cases show these mutations, indicating that other genetic factors may also contribute to the development of FAVA (9).

The analysis of PIK3CA's role in FAVA reveals both clinical and pathological implications. While PIK3CA mutations are prevalent in many cases, their presence does not always correlate with specific clinical features or outcomes. For instance, some patients without detectable PIK3CA mutations still exhibit similar clinical manifestations as those with the mutation (6, 9). Furthermore, immunohistochemical studies have shown activation of downstream effectors such as AKT and mTOR in lesions harboring PIK3CA mutations; however, these pathways may also be activated through alternative mechanisms in wild-type cases (8). This complexity underscores the need for further research into additional genetic alterations that could influence disease presentation and treatment responses.

Consistent with these molecular alterations, characteristic imaging findings are observed in FAVA. The imaging examinations of FAVA include MRI, color Doppler endoscopic ultrasonography, angiography, etc. Amarneh et al. (10) analyzed the MRI data of 38 FAVA patients were analyzed to conclude 3 MRI morphology features: 1. local mass, lesions involving a single anatomical region with over 75% of clear boundaries, and length, width, and height were all easy to measure; 2. focal infiltrative lesions with ill-defined demarcation, lesions involving a single anatomical region; at least one or two dimensions of length, width, and height can be easily measured; 3. a diffusely infiltrating lesion with unclear boundaries and involving one or multiple anatomical regions, and the lesion can not be precisely measured via imaging. Although there were differences in morphology and distribution, all MRI images of FAVA lesions exhibited inhomogeneous hyperintense signals of adipose tissue within involved muscles on T1WI, and signal intensities on T2WI were stronger. Some patients may manifest subcutaneous adipocyte hyperplasia (1). Ultrasonography and venography are also diagnostic measures for FAVA. Ultrasound findings of FAVA showed reticular veins and balloon-shaped dilatation in veins, mixed with varying degrees of inhomogeneous hyperechoic tissue. Hyperechogenicity might be related to lesions in the fibrous tissue in FAVA. Venography within FAVA lesions manifests extrafascial and intramuscular venous distensibility (string of beads) with slow blood flow.

The pathological findings of FAVA patients are homogeneous. Skeletal muscle, veins, fat, fibrous tissue, and lymphocyte aggregation were observed in HE staining (10, 11).

The differential diagnosis of FAVA includes venous malformation, arteriovenous malformation, soft tissue mass, etc. (12). The MRI image of FAVA lesions is characterized by fat infiltration, venous distensibility in the lesion, and subcutaneous venous distensibility or adipocyte hyperplasia. Since there is blood flow, thrombi, or phlebolith in the lesion, the MRI images of FAVA and other vascular malformations may all present inhomogeneous enhanced signals on T1WI. However, fat infiltration in FAVA lesions leads to artifacts on T2WI, while venous malformations present more homogeneous signals (10). Meanwhile, there are no sex differences in venous malformation onset and those patients usually don't have typical symptoms like pain, which is a key feature to distinguish FAVA from venous malformation (13). Klippel–Trenaunay Syndrome (KTS) manifests diffuse capillary malformations in the affected limb, disproportionate soft tissue hyperplasia, diffuse low-flow venous malformation, diffuse micro-capsules, and macrocystic lymphatic malformation (14). CLOVES syndrome manifests diffuse capillary malformation, adipocyte hyperplasia in the extremity/trunk, complex venous and lymphatic malformation, skeletal malformations, epidermal nevus, scoliosis, etc. (15). Compared with typical KTS and CLOVES syndrome cases, few cases of FAVA were reported to have capillary malformations nor soft tissue hyperplasia.

Luks et al. (12) confirmed PIK3CA mutations in FAVA patients through targeted genetic analyses, aligning with the gene's established functions in regulating cellular growth, survival, and metabolic processes. Recent studies have also identified somatic mutations in the PIK3CA gene as a key molecular feature in FAVA, implicating its role in pathogenesis through mTOR pathway activation. These gain-of-function mutations drive abnormal proliferation of vascular and adipose tissues, evidenced by immunohistochemical markers of mTOR signaling hyperactivity (6, 16). While PIK3CA mutations are recognized as the second most frequent genetic alteration in sporadic venous malformations (17), the molecular landscape of FAVA remains comparatively understudied. Notably, the therapeutic potential of PIK3CA inhibitors—previously validated in PIK3CA-driven disorders like Klippel-Trenaunay syndrome and CLOVES syndrome (18)-highlights the need to clarify mutation-specific mechanisms in FAVA. Further research is required to delineate how PIK3CA dysregulation uniquely contributes to FAVA's distinct clinico-pathological features compared to conventional venous malformations.

In the aspect of treatment, sclerotherapy has attained favorable efficacy in treating venous malformation (19, 20), but it can only control the symptoms of FAVA patients in a short term, presenting non-ideal efficacy (21). In a study involving 27 patients with FAVA, sclerotherapy was utilized in 11 cases (40.7%) without symptomatic improvement (8). Notably, while some patients reported transient pain relief, others experienced worsening symptoms or increased contractures post-treatment (6), highlighting the unpredictable response to sclerotherapy. The failure of sclerotherapy in FAVA may stem from: 1. Anatomic complexity—the fibrovascular-adipose architecture limits homogeneous drug distribution; 2. molecular resistance—activation of the PI3K-AKT-mTOR pathway may promote fibroadipose proliferation and reduce drug penetration (7, 9); 3. clinical heterogeneity—individual variability in lesion location and symptomatology necessitates patient-specific strategies.

In this case, histology confirmed dominant fibroadipose components (Figure 3), providing a structural basis for sclerotherapy failure. Shaikh et al. (3) performed color Doppler or CT-guided percutaneous cryoablation for 20 FAVA patients and found that this operation can improve pain and function restriction to a certain degree. However, cases included in the study were limited with short follow-up duration. The efficacy of percutaneous cryoablation still needs further validation. A recent study reported that sirolimus treatment led to significant tumor shrinkage and pain relief in patients with FAVA, highlighting its potential as a safe and effective adjunct therapy (22), but the efficacy of sirolimus remained to be explored long-term because of the small sample size. Considering the limitations of therapies described above, some scholars proposed that surgery alone can obtain favorable efficacy since FAVA is a relatively local solid lesion. But close attention must be paid to vessels and nerves, which should be carefully isolated to avoid injury (23). The surgical modality includes subtotal resection and total resection of FAVA lesions, neurolysis, and capsulotomy. Tendon lengthening is performed if necessary (23). Surgical techniques have evolved, and minimally invasive approaches are increasingly being adopted, allowing for reduced recovery times and improved cosmetic outcomes. For example, hybrid treatments combining ethanol sclerotherapy with surgical excision have shown promising results in managing FAVA lesions, demonstrating the potential for enhanced recovery and patient satisfaction (24).

Taken together, FAVA lesions are more common among young females, and the most frequent site of onset is the calf, mainly involving the gastrocnemius and soleus muscles. Acute pain after activities is one of the clinical features of FAVA, and some patients may show joint contracture. MRI, color Doppler, and venography are conducive to FAVA diagnosis, and currently, surgery alone is the preferred treatment modality for FAVA. As the understanding of the molecular biological mechanism of FAVA deepens, more precise and effective treatments will become available.

## Conclusion

Given the rarity of FAVA in the upper limb, there were no established guidelines for the treatment. Our described procedure led to complete resolution of the patient's symptoms. This demonstrates that surgery can be an acceptable solution to similar cases in appropriate surgical candidates.

## Data Availability

The original contributions presented in the study are included in the article/Supplementary Material, further inquiries can be directed to the corresponding author.
